# Significant common environmental effects on leukocyte subpopulations

**DOI:** 10.1371/journal.pone.0196193

**Published:** 2018-04-24

**Authors:** Maya Botzman, Irit Gat-Viks

**Affiliations:** Department of Cell Research and Immunology, The George S. Wise Faculty of Life Sciences, Tel Aviv University, Tel Aviv, Israel; Westmead Institute for Medical Research, AUSTRALIA

## Abstract

Major efforts are invested in the analysis of phenotypic variation in a population of individuals. While many of these studies focus on the genetic basis of phenotypic variation via measurements of DNA polymorphic sites, the environmental effects are still elusive. Here we propose a methodology, called CCCE ('Cell Composition Common Environment'), to identify environmental effects on the composition of immune cell functionalities. Specifically, CCCE is focused on the common experiences that are shared between siblings (the ‘common environment’), designed to correct for cell subpopulation heterogeneity, and is based on a multicolor flow cytometry analysis across a large cohort of human monozygotic and dizygotic twins. We demonstrate that the CCCE methodology can provide insights on the relations between common environmental effects and the heterogenic functions of several immune cell types, such as NK cells effector functions and coagulation-related capabilities of monocytes. The software described in this article is available at http://csgi.tau.ac.il/CCCE.

## Introduction

Trait variation is a result of both genetic and life history differences between individuals. The life experience of individuals, referred to as the ‘environmental effect’, has two main components: first, the common experiences shared between siblings that grow up in the same household (e.g., similar maternal care), referred to as the *common environmental effect*, and second, the specific experiences, which are unique to each individual, including biological and technical noise [1].

Common environmental effects, such as the same intrauterine experience and maternal care, diet and exposure to air pollution in shared indoor housing, are of particular interest in analyzing phenotypic variation, as they often convey important influences on early development [1]. In addition, the common environment also dictates the exposure to pathogens and immunization during early childhood, thus bearing possible implications on immune homeostasis and a variety of immune-related diseases [2]. Understanding the possible effects of the common environment on specific phenotypes is of fundamental interest and could facilitate the development of strategies for preventing and reducing environmental risks.

Immune homeostasis is an attractive system for the analysis of the environmental effects on global regulation of normal physiological conditions. Many aspects of immune cell physiology, such as proliferation and differentiation, can be revealed based on the quantity of different cell types. Recent immunological studies of twins examine the contribution of genetic and environmental effects to immune traits variation, and suggest that a substantial proportion of the variation in homeostatic cell quantities in the blood is due to non-genetic factors [3–7]. Moreover, the variation attributed to the common environment appears in a larger number of cell subpopulations than previously assumed [8]. Importantly, any protein presented on the cell surface likely performs part of a certain functionality of the cell, either the main cell functionality, or alternatively, a secondary or a subsidiary functionality. We therefore refer to any such protein in the context of a specific cell type as a *subfunction*. For instance, monocytes presenting the cell surface marker Thrombomodulin (THBD) carry the anticoagulation subfunction, in addition to their main role in innate and adaptive immunity [9]. Notably, recent work has demonstrated genetic effects acting on immune subfunctions [10]. Yet, despite the importance of such variation, we currently lack an assessment of the role of the environment in determining the composition of immune subfunctions during homeostasis.

Advances in flow cytometry analysis have opened the way to comprehensive multi-parametric analysis of immune cell subfunctions, facilitating quantification and isolation of the various cell types [11]. In addition, multicolor flow cytometry allows monitoring the expression of a panel of markers on the surface of cells as a rich annotation of various subfunctions in each individual single cell [12]. A typical multicolor flow cytometry, performed across a large cohort of individuals, can be therefore used to assess the magnitude of common environmental effects on immune cell subfunctions.

Here, we developed a computational approach, referred to as CCCE ('Cell Composition Common Environment'), which aims to identify common environmental effects on immune subfunctions. Whereas the standard approach investigates the inter-individual variation in the composition of immune cell subpopulations [8], CCCE identifies environmental effects on the composition of immune cell subfunctions while accounting for the heterogeneity in cell subpopulations. Notably, CCCE only relies on multicolor flow cytometry measurements across a large cohort of human twins, without requiring any prior knowledge or measurements of the underlying environmental factors.

Using CCCE, we demonstrate the existence of a common environmental effect on the distribution of subfunctions in four cell types (monocytes, effector NK cells, CD4^+^CD8^+^ αβ T cells and CD8^+^ T cells), highlighting core cell surface markers that likely relate to these common environmental effects. Our CCCE analysis further suggests that the environmental effects on the composition of cell subpopulations may fail to describe environmental effects on the composition of cell subfunctions. Taken together, our study highlights the importance of probing the effect of the common environment on immune subfunctions, even when knowledge about the underlying environmental factors is lacking.

## Methods

### Input data

The input dataset used by the CCCE algorithm is a collection of multicolor flow cytometry cell quantities, measured in a given cell type across a large cohort of monozygotic and dizygotic twins. The data is collected for each individual separately in two steps: first, one particular cell type is isolated using flow cytometry; and secondly, the isolated cells are analyzed using multicolor flow cytometry that simultaneously measures the level of several cell surface proteins in each single cell. We hereby refer to the set of cell surface markers analyzed simultaneously as a *panel*. Altogether, each dataset refers to a single cell type and a single panel of proteins whose cell surface level is measured by multicolor flow cytometry across a cohort of twins.

**Fig 1:**
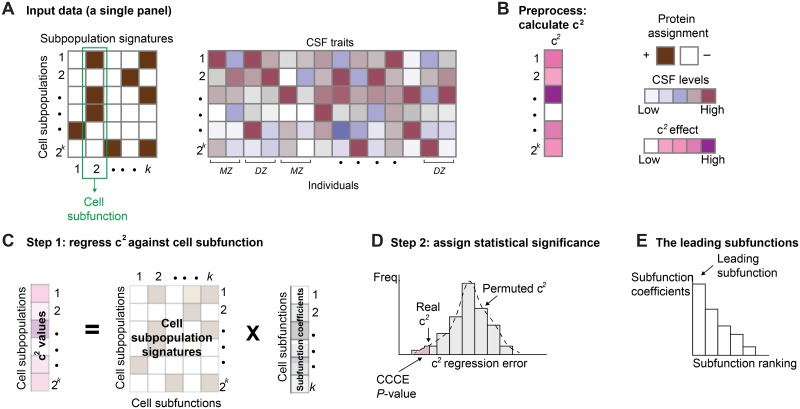
Overview of the CCCE method. (**A**) The input data, consisting of cell subpopulation signatures shown across the cell subfunctions (left), and the CSF traits of each subpopulation across dizygotic and monozygotic twins (right). (**B**) The pre-processing step, presenting the common environment effects for each cell subpopulations, calculated using the Falconer’s formula. (**C**) CCCE step 1. Regression of the common environmental effects using the cell functions as predictors. (**D**) CCCE step 2. A plot of the distribution of permutation-based prediction errors compared to the actual prediction error, providing a statistical significance score. (**E**) The leading subfunction. Shown are the resulting regression coefficients of each subfunction, highlighting the leading subfunction. Abbreviation: c^2^—the common, non-age-related, environmental effect.

Typically, a flow cytometry ‘gating criterion’ is a discretization of the cells into two groups based on the level of their cell surface proteins, either above (+) or below (-) a certain cutoff. In accordance, a *cell subpopulation* is defined as a subset of cells (of the same cell type) carrying the same discretized levels across the entire pre-defined panel of proteins (Fig 1A, **left**). In particular, a panel of *k* proteins defines 2^*k*^ cell subpopulations, where each such subpopulation is a group of cells carrying the same vector of discrete protein assignments x element of {+, -}^*k*^. We next define the *cell subpopulation frequency* (in short, CSF) as the ratio between the number of cells of a given cell subpopulation and the total number of cells of the same cell type. Such a measure is calculated independently for each cell subpopulation and for each individual. More globally, we further define the *CSF trait* as the CSF vector across all individuals (Fig 1A, **right**).

Overall, the CCCE input dataset is a collection of 2^*k*^ different CSF traits measured using a certain *k*-proteins panel (and its accompanying discretization cutoffs) in a specific cell type. Each trait encompasses individuals of both dyzygotic and monozygotic twin pairs (Fig 1A).

We note that each particular cell surface protein contributes to specific cellular subfunctions. In accordance, the term *cell subfunction* reflects the existence of one particular protein on the cell surface of a given cell type, regardless of the combination with any other cell surface protein (Fig 1A, **left**). Throughout this study we therefore distinguish between two interrelated terms: whereas a ‘cell subpopulation’ refers to a group of cells carrying the same combination of protein markers, a ‘cell subfunction’ refers to the functionality of a single protein, which may appear in many different cell subpopulations.

### Overview of CCCE

The CCCE input is a single dataset consisting of a collection of CSF traits for a single cell type (that is, a single flow cytometry panel) across the individuals participating in the study (all monozygotic and dizygotic twins). Each of the traits is accompanied by its corresponding signature of cell subfunctions (Fig 1A). Given these inputs, the algorithm aims to identify common environmental effects on specific cell subfunctions. Our rationale is that calculations of common environmental effects on the frequencies of cell subfunctions may lead to false positive predictions due to confoundings related to imbalance in cell subpopulation frequencies. For example, assume a highly prevalent cell subpopulation A that carries a cell surface marker *x*. Further assume that marker *x* resides on the cell surface of several rare subpopulations in the same tissue. We consider a scenario in which the common environmental effect acts only on the frequency of subpopulation A and has no effect on any other subpopulation. Due to the high prevalence of type-A cells in the data, it may be erroneously determined that the common environmental effect acts on the presence of marker *x* (subfunction x) rather than on the cell subpopulation A. To discriminate between these possibilities, CCCE evaluates the relations between the common environment and cell subfunctions while eliminating potential biases due to subpopulation-specific evidence.

In particular, CCCE first utilizes standard methods to calculate the common environmental effect for each cell subpopulation (Fig 1B). Next, CCCE aims to assess the ability of the various cell subfunctions to predict the common environmental effect, using a regularized regression framework and assuming an unbiased evidence from the different cell subpopulations (Fig 1C). Using permutations, CCCE determines the statistical significance of the relation between the immune subfunctions and the common environmental effects (a *P*-value score, Fig 1D). It may also be useful to extract the dominant subfunction that contributes to these relationships (Fig 1E). The CCCE algorithm proceeds as follows:

### Preprocessing step: Calculating the common environmental effect

Many computational methodologies aim to assess the statistical significance of the impact of a given (common or non-common) environmental factor on the phenotypic outcome [13]. These methods provide important insights regarding the effect of environmental factors, but require a-priori information about the putative environmental factor. However, the environmental factor is not always known a-priori. Even when the environmental factor is known, its monitoring still requires intensive labor and time (e.g., [14]). In addition, many environmental factors, such as the actual exposure to pathogens during childhood, are impractical to monitor.

To address these difficulties, an alternative strategy would be to avoid monitoring the environmental factors. In particular, the contribution of the common and non-common environmental effects on the total phenotypic variation is assessed solely based on the phenotypic diversity, using the Falconer’s formula [1]. In brief, the Falconer’s formula utilizes the difference between monozygotic and dizygotic twins to decompose the total trait variation (V_p_) into three types of variation: genetic heritability (*h*^*2*^), common environment variation (*c*^*2*^) and non-common environment variation (*e*^*2*^), through the equation: V_p_ = h^2^ + c^2^ + e^2^ (it is possible to add additional effect types such as gender). The calculation is based on the phenotypic differences between monozygotic twins, who share their genetic makeup and are exposed to the same common environment, versus dizygotic twins, who are also exposed to the same common environment but share only about 50% of their genetic makeup. Specifically, *h*^*2*^ = *2*(*r*_*mz*_ − *r*_*dz*_), *c*^*2*^ = *r*_*mz*_ *− h*^*2*^ = *2r*_*dz*_ − *r*_*mz*_, and *e*^*2*^ = *V*_*p*_ − *h*^*2*^ − *c*^*2*^, where *r*_*mz*_ is the trait’s correlation between the monozygotic twins, and *r*_*dz*_ is the trait’s correlation between the dizygotic twins. The Falconer formula thus allows evaluation of the common environment effect solely based on phenotypic variation in dizygotic and monozygotic twins, without requiring direct environmental measurements.

CCCE assumes a single common environmental effect acting on each of the cell subpopulations. The common environmental effects were calculated using the Falconer formula as described in Roederer et al. [8] (Fig 1B). Briefly, the calculation involves two steps: first, evaluating the age effect, estimated based on a linear least-squares fit of the CSF trait value by age, and then adjusting the trait for the confounding effect of individual age. Second, for each non-age-related CSF trait, the Falconer’s formula calculates the genetic and environmental effects. We hereby denote by c^2^ the common, non-age-related, environmental effect (c^2^ values were downloaded from [8]).

### Step 1: Identify common environment effects on cell functions

CCCE aims to calculate the extent to which the cell subfunctions are related to the heterogeneity of the common environmental, non-age-related effects (c^2^) across cell subpopulations. Naively, this can be done by calculating the c^2^ values for single-marker subpopulations (as if the panel consists of only one marker). However, such an approach is prone to false positive results due to the internal composition of the subpopulations. To tackle this, CCCE assumes that if a certain subfunction is affected by the common environment, a similar effect would be observed in all subpopulations carrying this subfunction. To avoid multiple testing of each individual subfunction, the CCCE model encompasses all subpopulations of a given isolated cell type into a single multiple linear regression model. In this regression model, the dependent variable is the common environmental effect (c^2^) and the *k* predictors are the collection of cell subfunctions (+1 if the corresponding protein is present, otherwise -1) across all 2^*k*^ subpopulations (CSF traits) of the same panel (Fig 1C). By solving the regression jointly for all cell subpopulations, the model provides optimized coefficients that are shared among all cell subpopulations. The CCCE model is therefore substantially different from the Falconer formula that is solved independently for each cell subpopulation.

To avoid overfitting, we used regularized multiple regression framework (here, elastic net [15]). In order to obtain the best elastic-net parameters, we performed K-fold cross validation and chose the elastic net parameters that minimize the model error. The *K*-fold cross validation is a procedure in which the dataset is partitioned into K smaller subsets. For each such subset a model is trained using the other K-1 subsets and the remaining subset is used to calculate the model error. The output ‘prediction error’ of the model is the averaged error across the K trained models. This procedure is repeated for different elastic net parameters, and the set of parameters that minimizes the prediction error is chosen. Here we set K to be the number of cell subpopulations divided by 5, in order to ensure a large number of folds while maintaining at least five cell subpopulations in each fold.

### Step 2: Assigning statistical significance

To assign statistical significance to this measure, we make the most conservative permutation by breaking potential ties between the dependent and predictor variables while maintaining the internal structure within each of these components. In particular, CCCE created 1000 shuffled datasets, each of which consists of permuted values of the dependent variable c^2^. For each shuffled dataset, CCCE calculates the best set of elastic net parameters (and the corresponding prediction error). Finally, CCCE compares the 1000 shuffling-based prediction errors to the real prediction error (Fig 1D). This process allowed us to give a significance value for a model of a given cell type (a permutation-based *P*-value score).

### The leading subfunction

Multiple subfunctions (markers) were included in a cell type model, yet not all of them were related to or controlled by the common environment. The top-scoring (highest absolute correlation coefficient value) subfunctions are therefore useful for interpretation of the results, being the subfunction with the highest contribution to the model (Fig 1E). In particular, a positive (negative) coefficient links a surface marker presence (absence) with common environmental effect. We refer to the top-scoring subfunctions as ‘leading subfunctions’.

In this study our main biological interest was to identify common environmental effects related to the presence of cell surface markers (positive-coefficients). However, the methodology is general and can be applied not only on common environmental effects but also on additional effect types (e.g., heritability or age). Furthermore, the analysis of leading subfunctions is general and may refer to both positive and negative coefficients.

## Results

**Fig 2:**
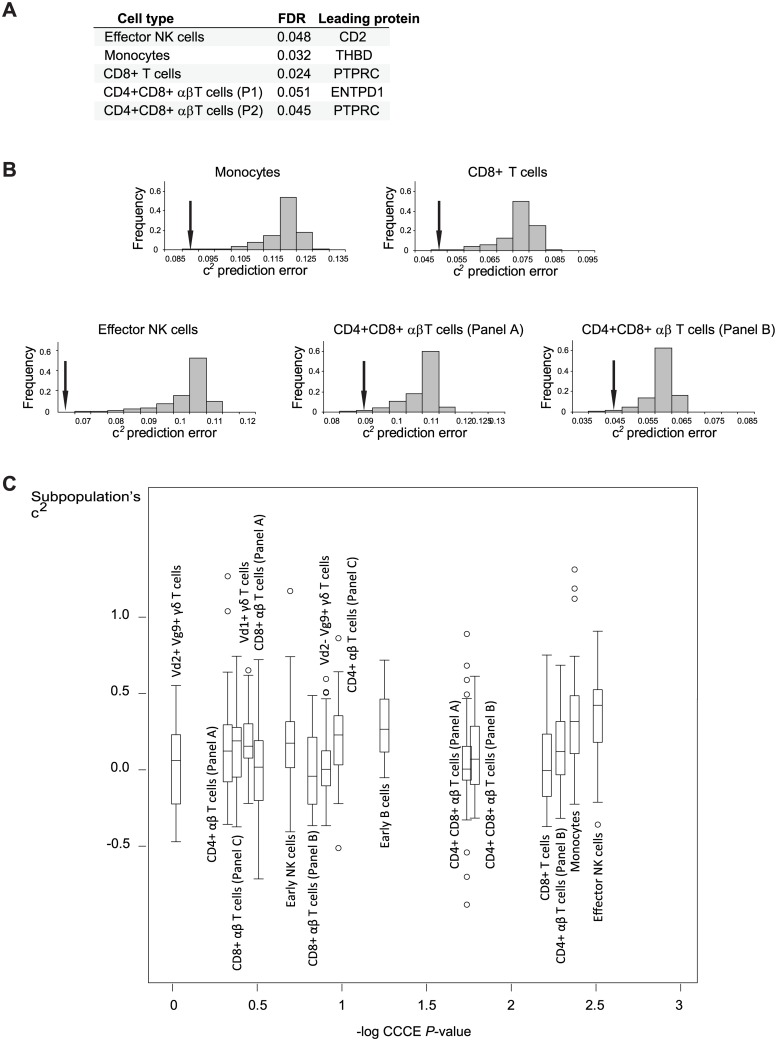
CCCE analysis of blood-derived leukocyte populations. (**A**) Cell types with significant CCCE *P*-values. The cell types, their FDR level according to the CCCE method and their leading proteins are shown. (**B**) The distribution of permutation-based regression errors (gray bars). The arrow indicates the point of the real-data prediction error. Plots for each significant CCCE score are presented (as reported in **A**), demonstrating their low errors compared to the expected (permuted) distribution. (**C**) Boxplots representing the common environmental effect on cell subpopulations in each cell type. The *x*-axis coordinate indicates the CCCE *P*-values (log scale). Abbreviation: c^2^—a common, non-age-related, environmental effect.

We applied the CCCE method on a collection of recently published datasets ([8]; downloaded from http://www.tinyurl.com/twinsFACSdata). These datasets were measured across 500 human female twins (156 monozygotic and 344 dyzygotic twins) and encompass 32 different cell types with 3 to 8 proteins in a flow panel for each cell type. As previously proposed [8], we included in our analysis only high-quality CSF traits exhibiting high replicate correlation (>0.3) and mean trait value ranging between 1% to 99%. As a result, we applied our analysis on 16 datasets with at least 18 of high-quality CSF traits (S1 Table). Fig 2A lists the identified datasets with significant CCCE P-values (FDR < 0.1): effector NK cells, monocytes, CD4^+^CD8^+^ αβ T cells [panels A and B] and total CD8^+^ T cells. For each of these datasets, Fig 2B demonstrates the CCCE calculation of statistical significance. As expected, CSF traits in significant datasets do include, but are not limited to, high common environmental effects (Fig 2C).

**Fig 3:**
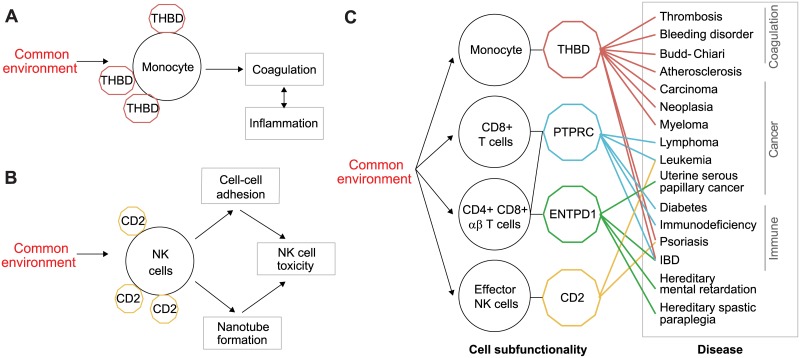
An integrated model of environment-subfunction-disease relationships. A network view of cell types with significant common environmental effect on the composition of subfunctions (Fig 1A, circles), each linked to the leading subfunction on which the environmental effect is exerted (hexagon). Based on the literature, each subfunction protein is further connected to certain diseases (rectangles). Whereas plots **A** and **B** provide zoom-in on the subnetwork of monocytes and effector NK cells, respectively, plot **C** shows the entire connectivity network.

Characterization of the leading cell surface proteins provide insights into the cell subfunctions that are likely affected by the common environment. For instance, the leading protein of the monocytes dataset is Thrombomodulin (THBD), an important thrombin cofactor involved in reduction of blood coagulation and regulation of inflammation [16,17]. Although monocytes primarily participate in the innate immune response, they have an additional subfunction in thrombosis-related processes [9,18]. Specifically, monocytes contribute both as procoagulants (for example in the presence of LPS or other infective stimulation) and as inhibitors of coagulation via their cell surface Thrombomodulin [9,18]. Taken together, our results indicate that the balance of the coagulation process and its coupling with inflammation through monocytes are affected by common environmental factors (Fig 3A).

The analysis further suggested an effect of the common environment on subfunctions of effector NK cells. The leading protein of NK cells was CD2, which plays important roles in NK cell activation by cell-cell adhesion to the target cell, leading to elimination of infected cells [19] (Fig 3B). An example for such cell-cell adhesion is the interaction between CD2 residing on the NK cell and CD58 and CD48 on the target cell [20]. This interaction also has an effect on the activation and regulation of NK cytotoxic activity [21]. In addition, CD2 expression is essential to the formation of nanotube between the NK cell and the target cell [21], thereby establishing a physical contact between the cells that enables a selective intercellular trafficking of vesicles, further influencing the NK cell toxicity [22,23].

The leading T cells proteins also have known important subfunctions. In particular, in both CD8^+^ T cells (activated T_c_ cells) and CD4^+^CD8^+^ αβ T cells [Panel B] (resting or naïve T cells), CD45RA (PTPRC) was found to be the leading protein. The subfunction of PTPRC, a tyrosine phosphatase, is regulation of T- and B-cell antigen receptor signaling, JAK kinases and cytokine receptor signaling [24]. We refer to this result as a positive control, as it is expected that stimulations from the environment influence the balance between naïve and memory T cells. The analysis further suggested CD39 (ENTPD1), a nucleotide metabolizing surface enzyme that is likely involved in T cell activation, as the leading protein of CD4^+^CD8^+^ αβ T cells [Panel A] [25].

## Discussion

The environmental factors influencing the immune system are diverse and might include diet [26], physical activity [27], smoking habits [28] and air pollution [29]. Notably, many of the factors are not yet known. Our goal was to explore common environmental factors and identify significant effects on the composition of cell subfunctions. Specifically, we aimed to assign statistical significance to common environmental effects without prior knowledge about the actual environmental factors. We thus developed the CCCE methodology that is based on a dataset of multicolor flow cytometry measurements across a population (Fig 1). When applied on a collection of datasets, CCCE identified several cell types whose distribution of subpopulations is likely affected by the common environment, including effector NK cells, monocytes, CD4^+^CD8^+^ αβ T cells and total CD8^+^ T cells (Fig 2). The functionality of some of the associated leading proteins in the specific cell type context, such as THBD in monocytes and CD2 in NK cells, has been previously described (e.g., THBD in monocytes and CD2 in NK cells, Fig 3A and 3B).

Our results open the opportunity to build a model of the relations between common environmental effects and various diseases. To construct such a model, we focused on the cell types identified by CCCE as targets of the common environment, and highlighted the leading protein of each cell type. We further added diseases that are known to be related to the cell surface protein (Fig 3C). For instance, THBD was found to be involved in bleeding disorder [30], THBD mutations were found associated with thrombosis [31], and its level of expression was associated with cancer [32–34], atherosclerosis [35] and immune-related diseases [36,37]; the expression of CD2 was associated with psoriasis [37] and T-cell acute lymphoblastic leukemia [38]; changes in the regulation of PTPRC were also associated with Colitis, Crohn’s disease [39], diabetes [40] and many other disease phenotypes; and finally, ENTPD1 down-regulation was found to be associated with uterine serous papillary cancer [41], and different ENTPD1 mutations were found to be associated with Crohn’s disease [42], hereditary spastic paraplegia [43] and hereditary mental retardation [44].

The integrated model suggests cell subfunctions that might mediate the effect of the common environment on physiological disease traits. Notably, the effect of the environment on the same disease can be mediated through several distinct subfunction proteins (e.g., THBD and CD2 mediate psoriasis; Fig 3C). This suggests combinatorial regulation of the environment on phenotypic diversity through cellular functionality, and further emphasizes the importance of comprehensive studies to uncover an increasingly detailed map of disease-environment relationships.

## Supporting information

S1 Table Input datasets used in this study. The table shows the cell type name of each dataset (column 1), its panel proteins (column 2), the number of high-quality CSF traits that were included in the analysis (column 3), the FDR based on the CCCE *P*-values (column 4), and the leading subfunction (a protein symbol; column 5). Only datasets with more than 17 high-quality CSF traits were included.
(XLS)

## References

[1] Wedel I (1962) Falconer D. S.: Introduction to Quantitative Genetics. Oliver and Boyd, Edinburgh and London 1960; 365 S., 35 s. 140–141 p. http://doi.wiley.com/10.1002/bimj.19620040211.

[2] Salvetti M, Ristori G, Bomprezzi R, Pozzilli P, Leslie RDG (2000) Twins: Mirrors of the immune system. Immunol Today 21: 342–347. 10.1016/S0167-5699(00)01658-3 10871876

[3] Brodin P, Jojic V, Gao T, Bhattacharya S, Angel CJL, Furman D, et al (2015) Variation in the human immune system is largely driven by non-heritable influences. Cell 160: 37–47. Available: 10.1016/j.cell.2014.12.020. 25594173 PMC4302727

[4] Brodin P, Davis MM (2017) Human immune system variation. Nat Rev Immunol 17: 21–29. Available: http://www.ncbi.nlm.nih.gov/pubmed/27916977. 10.1038/nri.2016.125 27916977 PMC5328245

[5] Liston A, Carr EJ, Linterman MA (2016) Shaping Variation in the Human Immune System. Trends Immunol 37: 637–646. Available: 10.1016/j.it.2016.08.002. 27692231

[6] Carr EJ, Dooley J, Garcia-Perez JE, Lagou V, Lee JC, Wouters C, et al (2016) The cellular composition of the human immune system is shaped by age and cohabitation. Nat Immunol 17: 461–468. Available: http://www.nature.com/doifinder/10.1038/ni.3371. 26878114 10.1038/ni.3371 PMC4890679

[7] Mangino M, Roederer M, Beddall MH, Nestle FO, Spector TD (2017) Innate and adaptive immune traits are differentially affected by genetic and environmental factors. Nat Commun 8: 13850 Available: http://www.nature.com/doifinder/10.1038/ncomms13850. 28054551 10.1038/ncomms13850 PMC5227062

[8] Roederer M, Quaye L, Mangino M, Beddall MH, Mahnke Y, Chattopadhyay P, et al (2015) The genetic architecture of the human immune system: A bioresource for autoimmunity and disease pathogenesis. Cell 161: 387–403. Available: 10.1016/j.cell.2015.02.046. 25772697 PMC4393780

[9] Satta N, Freyssinet JM, Toti F (1997) The significance of human monocyte thrombomodulin during membrane vesiculation and after stimulation by lipopolysaccharide. Br J Haematol 96: 534–542. 10.1046/j.1365-2141.1997.d01-2059.x 9054661

[10] Lu Y, Biancotto A, Cheung F, Remmers E, Shah N, McCoy JP, et al (2016) Systematic Analysis of Cell-to-Cell Expression Variation of T Lymphocytes in a Human Cohort Identifies Aging and Genetic Associations. Immunity 45: 1162–1175. Available: 10.1016/j.immuni.2016.10.025. 27851916 PMC6532399

[11] Herzenberg LA, Parks D, Sahaf B, Perez O, Roederer M, Herzenberg LA (2002) The history and future of the Fluorescence Activated Cell Sorter and flow cytometry: A view from Stanford. Clinical Chemistry. Vol. 48 pp. 1819–1827. 12324512

[12] De Rosa SC, Brenchley JM, Roederer M (2003) Beyond six colors: a new era in flow cytometry. Nat Med 9: 112–117. 10.1038/nm0103-112 12514723

[13] Patel CJ, Bhattacharya J, Butte AJ (2010) An environment-wide association study (EWAS) on type 2 diabetes mellitus. PLoS One 5 10.1371/journal.pone.0010746 20505766 PMC2873978

[14] Vrijheid M, Slama R, Robinson O, Chatzi L, Coen M, van den Hazel P, et al (2014) The human early-life exposome (HELIX): Project rationale and design. Environ Health Perspect 122: 535–544. 10.1289/ehp.1307204 24610234 PMC4048258

[15] Zou H, Hastie T (2005) Regularization and variable selection via the elastic net. J R Stat Soc Ser B Stat Methodol 67: 301–320. 10.1111/j.1467-9868.2005.00503.x

[16] Li Y, Kuo C, Shi G, Wu H (2012) The role of thrombomodulin lectin-like domain in inflammation. J Biomed Sci 19: 34 Available: http://www.jbiomedsci.com/content/19/1/34. 10.1186/1423-0127-19-34 22449172 PMC3342133

[17] Conway EM (2012) Thrombomodulin and its role in inflammation. Semin Immunopathol 34: 107–125. 10.1007/s00281-011-0282-8 21805323

[18] Shantsila E, Lip GYH (2009) The role of monocytes in thrombotic disorders: Insights from tissue factor, monocyte-platelet aggregates and novel mechanisms. Thromb Haemost 102: 916–924. 10.1160/TH09-01-0023 19888530

[19] Davis DM (2009) Mechanisms and functions for the duration of intercellular contacts made by lymphocytes. Nat Rev Immunol 9: 543–555. Available: http://www.nature.com/doifinder/10.1038/nri2602. 19609264 10.1038/nri2602

[20] McNerney ME, Kumar V (2006) The CD2 family of natural killer cell receptors. Curr Top Microbiol Immunol 298: 91–120. Available: http://www.ncbi.nlm.nih.gov/pubmed/16323413. 16323413 10.1007/3-540-27743-9_5

[21] Comerci CJ, Mace EM, Banerjee PP, Orange JS (2012) CD2 Promotes Human Natural Killer Cell Membrane Nanotube Formation. PLoS One 7 10.1371/journal.pone.0047664 23112830 PMC3480409

[22] Davis DM, Sowinski S (2008) Membrane nanotubes: dynamic long-distance connections between animal cells. Nat Rev Mol Cell Biol 9: 431–436. Available: http://www.nature.com/doifinder/10.1038/nrm2399. 18431401 10.1038/nrm2399

[23] Chauveau A, Aucher A, Eissmann P, Vivier E, Davis DM (2010) Membrane nanotubes facilitate long-distance interactions between natural killer cells and target cells. Proc Natl Acad Sci 107: 5545–5550. Available: http://www.pnas.org/cgi/doi/10.1073/pnas.0910074107. 20212116 10.1073/pnas.0910074107 PMC2851811

[24] Hermiston ML, Xu Z, Weiss A (2003) CD45: A Critical Regulator of Signaling Thresholds in Immune Cells. Annu Rev Immunol 21: 107–137. Available: http://www.annualreviews.org/doi/10.1146/annurev.immunol.21.120601.140946. 12414720 10.1146/annurev.immunol.21.120601.140946

[25] Dwyer KM, Deaglio S, Gao W, Friedman D, Strom TB, Robson SC (2007) CD39 and control of cellular immune responses. Purinergic Signal 3: 171–180. 10.1007/s11302-006-9050-y 18404431 PMC2096766

[26] Julia V, Macia L, Dombrowicz D (2015) The impact of diet on asthma and allergic diseases. Nat Rev Immunol 15: 308–322. Available: http://www.nature.com/doifinder/10.1038/nri3830. 25907459 10.1038/nri3830

[27] Gjevestad GO, Holven KB, Ulven SM (2015) Effects of Exercise on Gene Expression of Inflammatory Markers in Human Peripheral Blood Cells: A Systematic Review. Curr Cardiovasc Risk Rep 9: 34 Available: http://link.springer.com/10.1007/s12170-015-0463-4. 26005511 10.1007/s12170-015-0463-4 PMC4439514

[28] Evans DM, Frazer IH, Martin NG (1999) Genetic and environmental causes of variation in basal levels of blood cells. Twin Res 2: 250–257. Available: http://www.ncbi.nlm.nih.gov/pubmed/10723803. 10723803 10.1375/136905299320565735

[29] Huang S-K, Zhang Q, Qiu Z, Chung KF (2015) Mechanistic impact of outdoor air pollution on asthma and allergic diseases. J Thorac Dis 7: 23–33. 10.3978/j.issn.2072-1439.2014.12.13 25694815 PMC4311071

[30] Dai L, Mitchell M, Savidge G, Alhaq A (2004) The profibrinolytic effect of plasma thrombomodulin in factor XI deficiency and its implications in hemostasis. J Thromb Haemost 2: 2200–2204. 10.1111/j.1538-7836.2004.01034.x 15613027

[31] Kunz G, Öhlin AK, Adami A, Zöller B, Svensson P, Lane DA (2002) Naturally occurring mutations in the thrombomodulin gene leading to impaired expression and function. Blood 99: 3646–3653. 10.1182/blood.V99.10.3646 11986219

[32] De Leval L, Rickman DS, Thielen C, Reynies D, Huang Y, Delsol G, et al (2015) The gene expression profile of nodal peripheral T-cell lymphoma demonstrates a molecular link between angioimmunoblastic T-cell lymphoma (AITL) and follicular helper T (T FH) cells. 109: 4952–4964. 10.1182/blood-2006-10-055145 17284527

[33] Stearman RS, Dwyer-nield L, Zerbe L, Blaine SA, Chan Z, Bunn PA Jr, et al (2005) Analysis of Orthologous Gene Expression between Human Pulmonary Adenocarcinoma and a Carcinogen-Induced Murine Model. Am J Pathol 167: 1763–1775. Available: 10.1016/S0002-9440(10)61257-6. 16314486 PMC1613183

[34] Twine NC, Stover JA, Marshall B, Dukart G, Hidalgo M, Stadler W, et al (2003) Disease-associated Expression Profiles in Peripheral Blood Mononuclear Cells from Patients with Advanced Renal Cell Carcinoma. CANCER Res Clin Res Dev Wyeth Res 63: 6069–6075. 14522937

[35] Rabausch K (2004) Regulation of Thrombomodulin Expression in Human Vascular Smooth Muscle Cells by COX-2-Derived Prostaglandins. Circ Res 96: e1–e6. 10.1161/01.RES.0000153150.27690.f2 15591227

[36] Scaldaferri F, Sans M, Vetrano S, Graziani C, De Cristofaro R, Gerlitz B, et al (2007) Crucial role of the protein C pathway in governing microvascular inflammation in inflammatory bowel disease. J Clin … 117: 1951–1960. Available: http://www.jci.org/cgi/content/abstract/117/7/1951. 10.1172/JCI31027 PMC1884689 17557119

[37] Oestreicher J, Walters I, Kikuchi T, Gilleaudeau P, Surette J, Schwertschlag U, et al (2001) Molecular classification of psoriasis disease- associated genes through pharmacogenomic expression profiling. Pharmacogenomics J 1: 272–287. Available: www.nature.com/tpj. 11911124 10.1038/sj.tpj.6500067

[38] Arber DA, Orazi A, Hasserjian R, Thiele J, Borowitz MJ, Le Beau MM, et al (2016) The 2016 revision to the World Health Organization classi fi cation of myeloid neoplasms and acute leukemia. Blood 127: 2391–2406. 10.1182/blood-2016-03-643544 27069254

[39] Alkim C, Balci M, Alkim H, Dağli U, Parlak E, Tezel A, et al (2007) The importance of peripheral immune cells in inflammatory bowel disease. Turk J Gastroenterol 18: 82–88. 17602351

[40] Smerdon RA, Peakman M, Hussain MJ, Alviggi L, Watkins PJ, Leslie RD, et al (1993) Increase in simultaneous coexpression of naive and memory lymphocyte markers at diagnosis of IDDM. Diabetes 42: 127–133. 8420810 10.2337/diab.42.1.127

[41] Santin AD, Zhan F, Cane’ S, Bellone S, Palmieri M, Thomas M, et al (2005) Gene expression fingerprint of uterine serous papillary carcinoma: identification of novel molecular markers for uterine serous cancer diagnosis and therapy. Br J Cancer 92: 1561–1573. Available: http://www.pubmedcentral.nih.gov/articlerender.fcgi?artid=2362016&tool=pmcentrez&rendertype=abstract. 10.1038/sj.bjc.6602480 15785748 PMC2362016

[42] Ernst PB, Garrison JC, Thompson LF (2010) Much Ado about Adenosine: Adenosine Synthesis and Function in Regulatory T Cell Biology. J Immunol 185: 1993–1998. Available: http://www.jimmunol.org/cgi/doi/10.4049/jimmunol.1000108. 20686167 10.4049/jimmunol.1000108 PMC3036969

[43] Novarino G, Fenstermaker a G, Zaki MS, Hofree M, Silhavy JL, Heiberg AD, et al (2014) Exome sequencing links corticospinal motor neuron disease to common neurodegenerative disorders. Science (80-) 343: 506–511. Available: http://www.ncbi.nlm.nih.gov/pubmed/24482476. 10.1126/science.1247363 PMC4157572 24482476

[44] Najmabadi H, Hu H, Garshasbi M, Zemojtel T, Abedini SS, Chen W, et al (2011) Deep sequencing reveals 50 novel genes for recessive cognitive disorders. Nature 478: 57–63. Available: http://www.nature.com/doifinder/10.1038/nature10423. 21937992 10.1038/nature10423

